# A survey of protein interaction data and multigenic inherited disorders

**DOI:** 10.1186/1471-2105-14-47

**Published:** 2013-02-11

**Authors:** Antonio Mora, Katerina Michalickova, Ian M Donaldson

**Affiliations:** 1 Department for Molecular Biosciences, University of Oslo, P.O. Box 1041 Blindern, 0316, Oslo, Norway; 2 The Biotechnology Centre of Oslo, University of Oslo, P.O. Box 1125 Blindern, 0317, Oslo, Norway; 3 Scientific Computing Group, University of Oslo, P.O. Box 1059 Blindern, Oslo, Norway

## Abstract

**Background:**

Multigenic diseases are often associated with protein complexes or interactions involved in the same pathway. We wanted to estimate to what extent this is true given a consolidated protein interaction data set. The study stresses data integration and data representation issues.

**Results:**

We constructed 497 multigenic disease groups from OMIM and tested for overlaps with interaction and pathway data. A total of 159 disease groups had significant overlaps with protein interaction data consolidated by iRefIndex. A further 68 disease overlaps were found only in the KEGG pathway database. No single database contained all significant overlaps thus stressing the importance of data integration. We also found that disease groups overlapped with all three interaction data types: n-ary, spoke-represented complexes and binary data – thus stressing the importance of considering each of these data types separately.

**Conclusions:**

Almost half of our multigenic disease groups could potentially be explained by protein complexes and pathways. However, the fact that no database or data type was able to cover all disease groups suggests that no single database has systematically covered all disease groups for potential related complex and pathway data. This survey provides a basis for further curation efforts to confirm and search for overlaps between diseases and interaction data. The accompanying R script can be used to reproduce the work and track progress in this area as databases change. Disease group overlaps can be further explored using the iRefscape plugin for Cytoscape.

## Background

Disease definition and classification is an open problem and, nowadays, the traditional phenotype-based definitions and classifications such as the International Classification of Diseases (ICD-10) [1] or the Systematized Nomenclature of Medicine (SNOMED) [2] have been enriched with molecular-based classifications with roots in genetic association databases such as Online Mendelian Inheritance In Man (OMIM) [3] and Genetic Association Database (GADB) [4], biological network analyses [5-7] and over-representation engines such as the DICS (dense modules from protein interaction networks) repository [8] and DAVID [9,10].

As a sample of these works, Goh *et al*. [6] introduced the term “Diseasome” while generating a bipartite graph of all known genetic disorders (disease phenome) to the list of known disease genes (disease genome). They presented a “Disease Gene Network” (DGN) connecting genes related to the same diseases, and a “Human Disease Network” (HDN), connecting diseases related to the same gene. Lage *et al.*[7] introduced a network linking protein interactions to human disease groups generated by text mining techniques, in an attempt to re-define disease according to annotation in databases, and developed a predictor of genes to be considered as related to some diseases. Feldman *et al.*[5] generated their own disease-interactome network and examined the network properties of disease-related gene mutations, finding that genes with intermediate connectivity are more probably disease-related and that disease genes display significant functional clustering. Dietmann *et al.*[8] generated a web-based repository of protein complexes and computationally predicted functional modules, which allows the user to detect complexes or modules significantly enriched in a list of genes.

Most of the previous works start from protein interaction networks (PINs) constructed by merging a few popular databases without explicitly stating how redundant information is normalized (interaction records from different databases may support identical or similar protein interactions but using different identifiers or data models). In addition, some of these studies use mathematically generated “modules” that do not necessarily correspond to a biological concept or observation. These factors make it difficult to cross-compare strategies for examining relationships between diseases and interaction data.

In this paper, we present an analysis of the overlaps between disease groups and interaction data as well as pathway data. The work represents a baseline analysis that can be easily reproduced using the provided data sets and scripts. We use the iRefIndex as a source of interaction data consolidated from 13 different databases [11] where proteins are normalized to a canonical representative in order to limit redundancy (for example, by representing all splice-isoforms of a protein using a single representative) [12,13]. This redundancy issue is especially important given that we employ the hypergeometric test in order to assess overlap between disease groups and interaction data. In addition, we pay special attention to treating separately overlaps between disease groups and the three types of interaction data: from binary records, n-ary records and spoke-represented complex records [14]. We show that each is an important source of complementary data. Finally, we show how disease-interaction overlaps can be monitored from one release of iRefIndex to another as the underlying data sets grow. In this paper, we have restricted our analysis to a simple ad-hoc definition of disease groups – titles from OMIM records are grouped using regular expressions to identify highly-similar titles describing various sub-types of the same disease. The causative genes are assembled into a “disease group”. This is a conservative grouping method and is easily reproduced – the groups themselves are also provided in the additional material. Other, more sophisticated disease and phenotype groupings are possible and the provided groups of disease genes can be easily replaced for comparison purposes.

We focus on the relationship between disease groups and protein groups, such as complexes or pathways, to assess to what extent groups of genes that are phenotypically related are involved in the same protein group. Many known diseases are multigenic in nature and, in some cases, may encode different partners in the same interaction, complex or pathway; disruption of any of these may lead to a related phenotype [15,16]. One example is the disease known as “Cerebral Cavernous Malformations” (CCM). According to OMIM, there are three types of CCM: CCM1 has been related to the KRIT1 gene, CCM2 to the CCM2 (Malcavernin) gene and CCM3 to the PDCD10 gene. A search in iRefIndex reveals that these three genes belong to a single complex (IntAct ID = EBI-1573226). Therefore, a “CCM” disease group may be confidently related to one malfunctioning protein complex. In addition, some diseases have been reported to be related to dimers (for example, Retinitis pigmentosa and Nephrotic syndrome), ligand-receptor disruption (Hirschprung disease and Alagille syndrome) or pathway disruption (Congenital disorder of glycosylation) [17]. A more complicated example is “Fanconi Anemia”. The 13 genes causing Fanconi Anemia have been recently grouped into three categories [18]: 8 genes form the so-called “Fanconi Anemia core complex”, 2 genes belong to the “FA-ID complex”, 3 other genes act downstream as regulators, and all three groups have been called the “FA pathway”.

These cases represent known relationships between diseases and complexes (or pathways) and they are often cited in the literature as evidence supporting the idea that similar phenotypes may have underlying molecular associations. We wished to examine this idea for a spectrum of diseases in order to determine how broadly applicable it may be. In a previous paper, we introduced a plugin for Cytoscape (iRefScape [19]) that allows the user to search for interaction data related to any one of the disease groups used in this paper. Here, we examine how likely a user is to retrieve statistically significant results that may help shed light on the mechanism of a disease. We also lay the ground work to track the progress of this result set over time as disease-group and interaction data evolve.

## Results

We compared thirteen protein interaction databases (consolidated in iRefIndex) to groups of proteins that are assumed to be involved in related diseases, which we called “Disease Groups” (DiG), in order to computationally determine all known diseases that might be related to known protein complexes and interactions in humans. After that, we performed a similar analysis for pathway databases. All analyses, tables and figures can be reproduced using the accompanying R script (Additional file 1).

### Construction of disease groups

Human inherited phenotypes and their related genes found in the OMIM Morbid Map [20] were assembled into related groups based on their titles, as described in Methods. This method provided groups of genes that were used throughout the remainder of the study to identify related complexes and subnetworks found in protein interaction databases.

Each table-entry from the OMIM Morbid Map describes a potential causal relationship between some gene listed in Entrez Gene [21] and a disease listed in OMIM. We used a system of regular expressions to identify sets of OMIM disease titles that were either identical or similar in order to arrive at groups of genes that were associated with phenotypically related diseases. Each group of related genes was assigned a distinct Disease Group Identifier (DiG ID) using this method.

The OMIM Morbid Map contained 5504 entries. 4116 of these entries contained strong evidence (evidence code 3) for a relationship between a gene and a disease. These were assigned to 1585 distinct disease groups (DiG IDs) where each group contained between 1 and 59 genes. Only 497 disease groups contained two or more associated genes and Additional file 2 shows the distribution of genes for these multigenic disease groups is highly skewed (mean 5.33 genes per DiG, standard deviation 6.57, skewness 4.22, median 3, mode 2 genes per group). 94.4% of the multigenic DiGs (469) have between 2 and 14 genes per DiG. The most complex disorders, in terms of number of associated genes, are: Deafness (59), Mental retardation (52), Leukemia (45), Diabetes mellitus (44), Retinitis pigmentosa (43), Colorectal cancer (38) and Cardiomyopathy (37).

A total of 2437 human genes are involved in all disease groups and 1940 in multigenic groups (2 or more genes). The complete table of disease groups, their corresponding Entrez GeneIDs and OMIM identifiers are provided in Additional file 3. A summary table including gene and protein information per DiG is provided as Additional file 4. In this table, the DiG Name corresponds to the string identified by our regular expressions and, in most cases, can be used as a valid name for a disorder whose variants can be interpreted as specific disease subtypes corresponding to the distinct OMIM entries in the group; however, in a few cases, this string is only part of what should be a descriptive name of the inherited disorder, such as “viral infection”, which stands for “susceptibility to viral infection”, or “molybdenum cofactor”, which stands for “molybdenum cofactor deficiency”.

**Figure 1:**
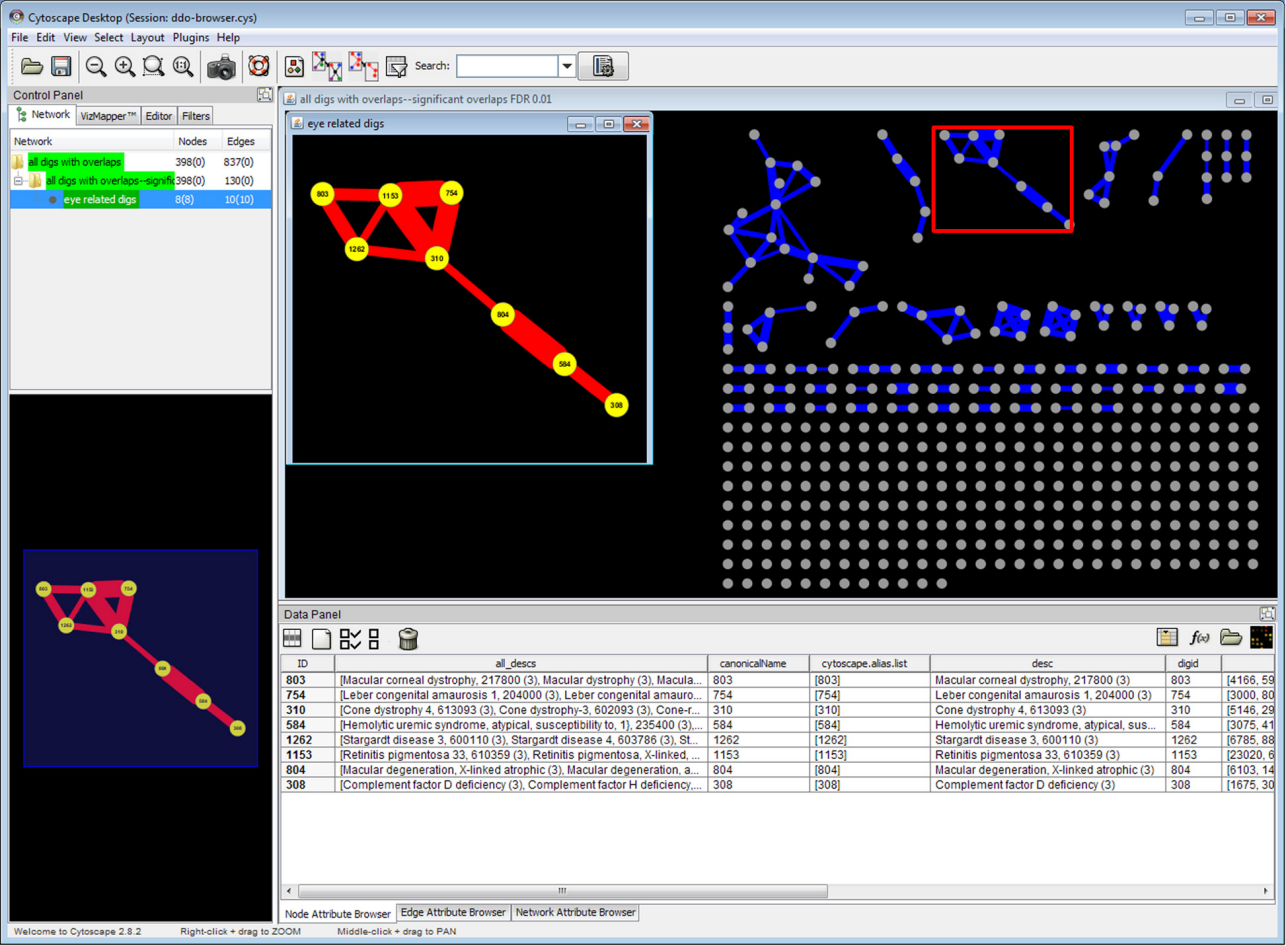
**Disease groups and their significant overlaps.** Cytoscape is used to visualize the disease groups and their overlaps. Additional file 5 can be directly loaded into Cytoscape to replicate the figure and explore the disease groups. Each node represents a group of related diseases associated with two to 59 genes. Edges represent one or more genes that are shared between disease groups where the width of the edge is proportional to the Jaccard index. The graph is sparse. Only 837 edges exist between the 497 multigenic disease groups and 130 of these overlaps are significant (hypergeometric test, p-value < 0.01 after FDR adjustment) – the above shows only these significant overlaps but all are available in the provided file. A number of connected components group together related disease groups such as cancer and eye disorders (red box magnified in inset).

Of course, some genes or even sets of genes are involved in multiple disease phenotypes; for example BRCA1 (Entrez GeneID: 672) is implicated in forms of both breast cancer and Fanconi Anemia. As such, our disease groups can be thought of as nodes in a graph where two disease groups share an edge if they share one or more genes. We examined these overlaps between disease groups and found that 398 of the disease groups share at least one gene with one other group 837 times and that 130 of these are significant (hypergeometric test, p < 0.01, false-discovery rate (FDR) adjusted). The resulting DiG network is a sparse graph composed mostly of isolated DiGs and a few larger components that contain DiGs related to such diseases as cancer and eye-disorders (Figure 1). These DiGs and their overlaps may be further explored using Cytoscape and Additional file 5. We show below that some protein complexes overlap with multiple disease groups when those disease groups are themselves closely related.

In a few cases, our disease grouping method failed to group together multiple OMIM entries and their associated genes because their titles are completely different. For example, Digenic iminoglycinuria (DiG 689) and Hyperglycinuria (DiG 631) both contain the same three genes. This complete overlap between two disease groups happens in only one other case. We found that OMIM entries describing phenotypically related diseases are named uniformly for the most part and decided that this regular expression method provided easily interpretable groupings sufficient for an initial survey of interaction data. These groups are provided as a baseline method but could be easily replaced with alternatives in future studies.

### Overlap between disease groups and n-ary data

The human network may be divided into two data types. Edges between protein nodes represent evidence for some relationship (direct or indirect, physical or genetic) between just those two proteins. We refer to these data as “binary” interaction records. In contrast, original source records from databases may list *three* or more protein interactors. In these cases, the experimental evidence is incapable of supporting a binary relationship between any given pair of proteins in the list; instead, the evidence simply supports the idea that all of the proteins are somehow related via some unknown set of direct or indirect binary interactions. The iRefIndex network contains a second node type to represent a complex of proteins. Edges between a complex node and a protein node represent membership of the protein in that complex. We refer to this data type as n-ary or “complex” data. A more detailed explanation of interaction data types used in this paper and their representation is included in the methods section along with a Figure at the end of this paper.

Source databases including this type of information for Homo sapiens include IntAct [22], HPRD [23], CORUM [24], DIP [25], BIND [26], MINT [27] and InnateDB [28], and, to this date, there are a total of 5677 distinct human complexes.

We assessed the overlap between each of our disease groups and each complex in the n-ary subset using a hypergeometric test and adjusting the p-values for multiple testing using the Bonferroni, FDR and BY methods (see Methods). From these three metrics, we chose FDR to identify the best match to the DiG, since it was the less conservative correction. Results for all DiGs are provided as Additional file 6.

This table shows that 94 DiGs were found to be significantly similar (p-value < 0.05, FDR adjusted) to at least one complex in the n-ary subset. That is, 19% of multigenic DiGs could potentially be explained as complexes where different malfunctioning subunits may give raise to different varieties of the disease.

DiGs can overlap with multiple n-ary records and we wanted to have a rough idea of the maximal number of n-ary complexes that an analyst might have to review when looking for overlaps with a disease group. DiGs can match at least one subunit of a number between 0 and 405 complexes (what we have named the “complex span” of a DiG). However, the significant matches (raw p-value < 0.05) are between 1 and 350, and the corrected matches (after the FDR adjustment) have a complex span between 1 and 40. The most complex disease in terms of overlapping complexes of the n-ary subset is colorectal cancer (complex span = 405 and significant matches = 40), followed by breast cancer (complex span = 270 and significant matches = 32). In these two cases, the number of records that would have to be reviewed is prohibitive. However, these are exceptions and 95% of all DiGs with significant overlaps match only 8 or fewer n-ary complexes.

**Figure 2:**
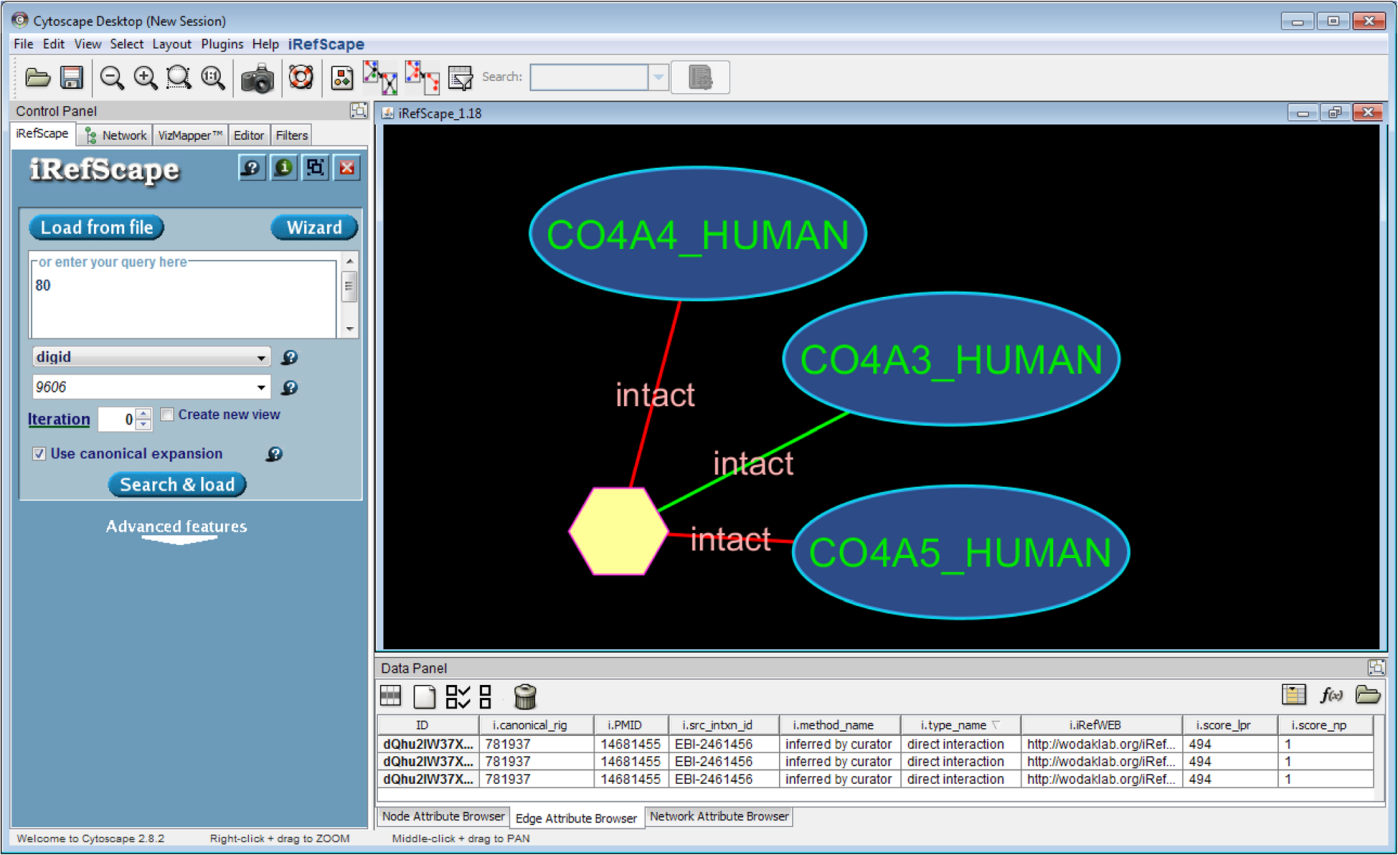
**Alport syndrome and subunits of type 4 collagen.** Alport syndrome is associated with three genes: COL4A5 with the X-linked form (MIM 301050) while COL4A3 and COL4A4 are associated with the autosomal-recessive form (MIM 203780). These forms were grouped into disease group 80. Searching for this disease group in iRefScape for interactions between any of the proteins in this disease group returns one record from IntAct (EBI-2461456) describing a complex (hexagon) containing all three proteins (ovals).

Figure 2 shows one such example. Alport syndrome is associated with three genes: COL4A5 with the X-linked form (MIM 301050) while COL4A3 and COL4A4 are associated with the autosomal-recessive form (MIM 203780). These forms were grouped into disease group 80 based on their similar OMIM titles. Searching for this disease group in iRefScape for interactions between any of the proteins in this disease group returns one record from IntAct describing a complex containing all three proteins (EBI-2461456). Alport syndrome is in fact a genetically heterogeneous disorder where all forms result from mutations encoding type IV collagen components which form a major structural component of the basement membrane. These relationships are noted in OMIM, however, there are no cross-references to interaction data nor is there a complete listing of all six type IV collagen isoforms (A1-A6) that are components of the type IV collagen superstructure. This structure is indeed implicated in Alport syndrome (as well as Benign familia hematuria) but the structure itself is quite complex (reviewed in [29]). Each of the type IV collagen isoforms can combine with other isoforms to form trimers: (A1/A1/A2) or (A3/A4/A5) or (A5/A5/A6). In turn, these heterotrimeric protomers can interact with one other protomer at their amino termini and three other protomers at their carboxy termini to form the supramolecular-mesh that is the structural component of type IV collagen. Alport mutations disrupt the formation of the A3/A4/A5 protomer network thus explaining the molecular etiology of the disease. The IntAct record (EBI-2461456) corresponds to a description of this protomer. The record contains no link back to the OMIM record although links between the disease and type IVcollagen in general could be indirectly made by examination of the underlying UniProt records.

While we found that 19% of our disease groups had a significant overlap with n-ary data, we feel that a manual review of existing overlaps and a search for new overlaps would be pre-requisite to reaching any quantitative conclusions about the relationship between multigenic diseases and protein complexes - a review of all significant disease-complex overlaps found in this study showed that no single database has systematically covered all diseases for potential overlaps with protein complexes (see below). So a manual review and search is required and this is non-trivial. For example, the work involved in confirming just the one overlap between Alport syndrome and the A3/A4/A5 protomer required a re-searching of the primary literature – the interaction record did not cite any experimental evidence and the listed PubMed identifier points to a publication for the IntAct database itself. This example is not isolated nor is the problem specific to any one database. The record in question pre-dates IMEx curation rules [30] and would not be accepted today as an official IMex record. However, these historical records and those produced by non-IMex member databases are an important source of disease-complex overlaps. A review of these data could help put in place curated, reciprocal links between disease and interaction databases and would facilitate future automated data mining efforts.

**Figure 3:**
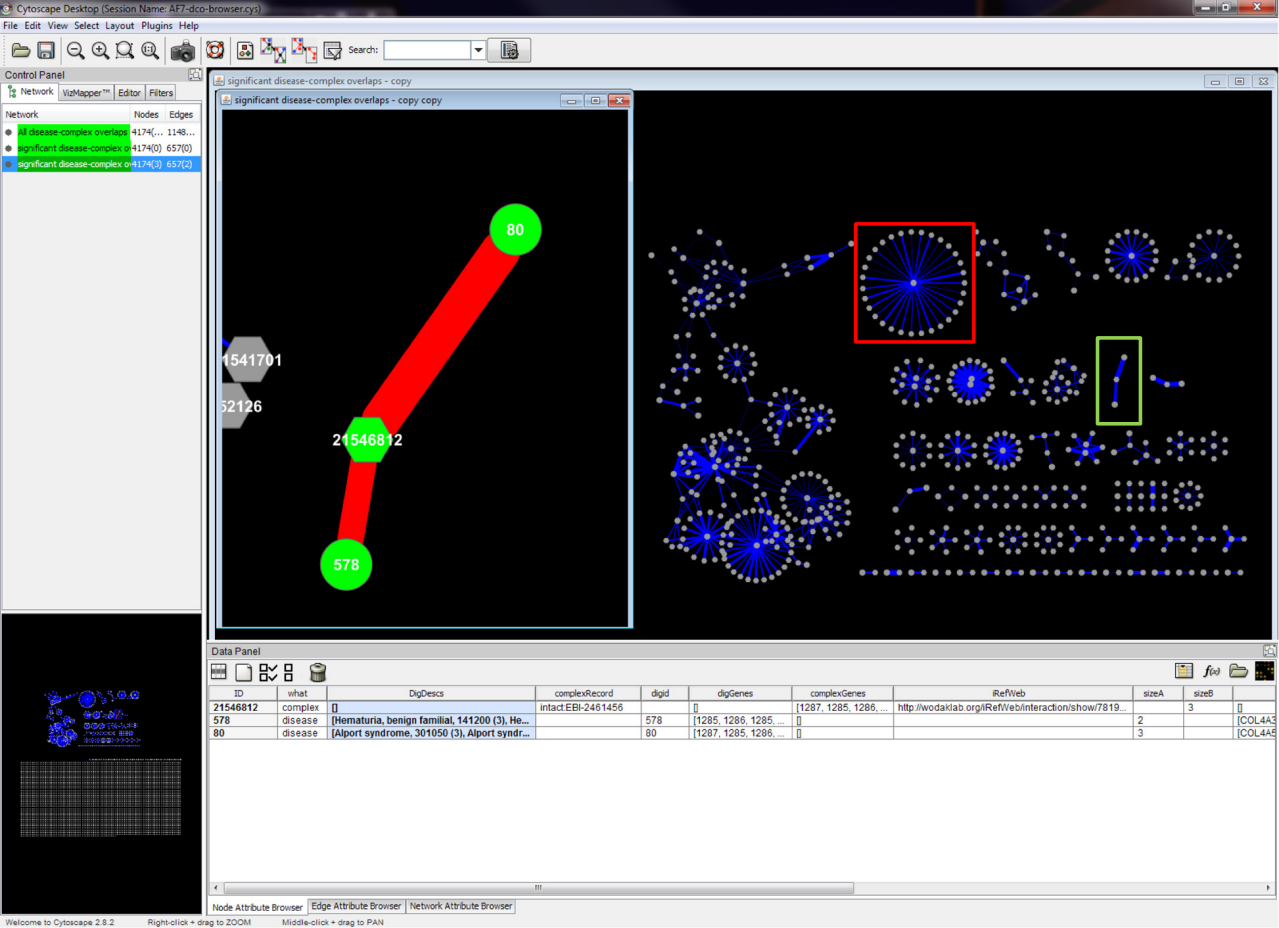
**Disease group overlaps with complexes.** Cytoscape is used to visualize disease groups and their overlaps with n-ary interaction records. Additional file 7 can be directly loaded into Cytoscape to replicate the figure and explore the overlaps. Disease groups (circular nodes) and n-ary records (hexagonal nodes) that have significant overlaps are indicated by edges whose width is proportional to the jacaard index [31] for the overlap. All disease-group overlaps with complexes are provided in the additional file. Here, significant overlaps (hypergeometric test, p-value < 0.0025 after FDR adjustment) are shown on the right and involve 105 disease groups. The region inside the red box shows an overlap between the Cornelia de Lange syndrome disease group and n-ary records that contain subunits of the cohesion complex. The region inside the green box (and magnified in the left inset) shows two disease groups (Benign familial hematuria and Alport syndrome) that both overlap with the same n-ary record (see text for details).

To facilitate this review process, we have included Additional file 7 that can be used to view all disease-complex overlaps in Cytoscape (Figure 3). A disease group may overlap with multiple observations of related n-ary complexes. For example, the region inside the red box shows an overlap between the Cornelia de Lange syndrome disease group and multiple n-ary records that contain subunits of the cohesion complex – all three causative genes in this disease group are members of the cohesion complex (MIM 122470). Conversely, a complex may overlap with multiple disease groups indicating a potential relationship between the diseases. For example, (in the green box) Benign familial hematuria (MIM 141200) and Alport syndrome (MIM 203780) are both renal diseases due to type IV collagen defects of the basement membrane. Both of these disease groups overlap with the collagen protomer described above and shown in Figure 2.

### Overlap between disease groups and regenerated n-ary data

In a previous work [14], we have shown that some primary databases represent complexes using a spoke model, making them virtually indistinguishable from binary data and, therefore, the iRefIndex contains information regarding complexes that would normally be treated as binary data. We developed an algorithm to extract this information as a set of “regenerated complexes”. This method is currently used by both iRefR and iRefScape to identify potential spoke-represented complexes [14,19]. Using “iRefR” with the software default values, 9947 potential spoke-represented complexes were regenerated (see Methods).

We assessed the overlap between each of our disease groups and each complex in the regenerated subset again using a hypergeometric test and adjusting the p-values for multiple testing using the Bonferroni, FDR and BY methods. Results for all DiGs are provided as Additional file 8.

This table shows that 110 DiGs were found to be significantly similar to at least one complex in the regenerated subset. That is, there is potentially more disease related information in spoke-represented complexes than there is in complex data represented as such. However, it is important to note that these “spoke-represented complexes” are only potential and it is possible that they may simply represent a set of unrelated binary interactions that, by chance, are observed in the same study using the same method and the same bait. A re-reading of the original source paper is required to distinguish between these two cases.

**Figure 4:**
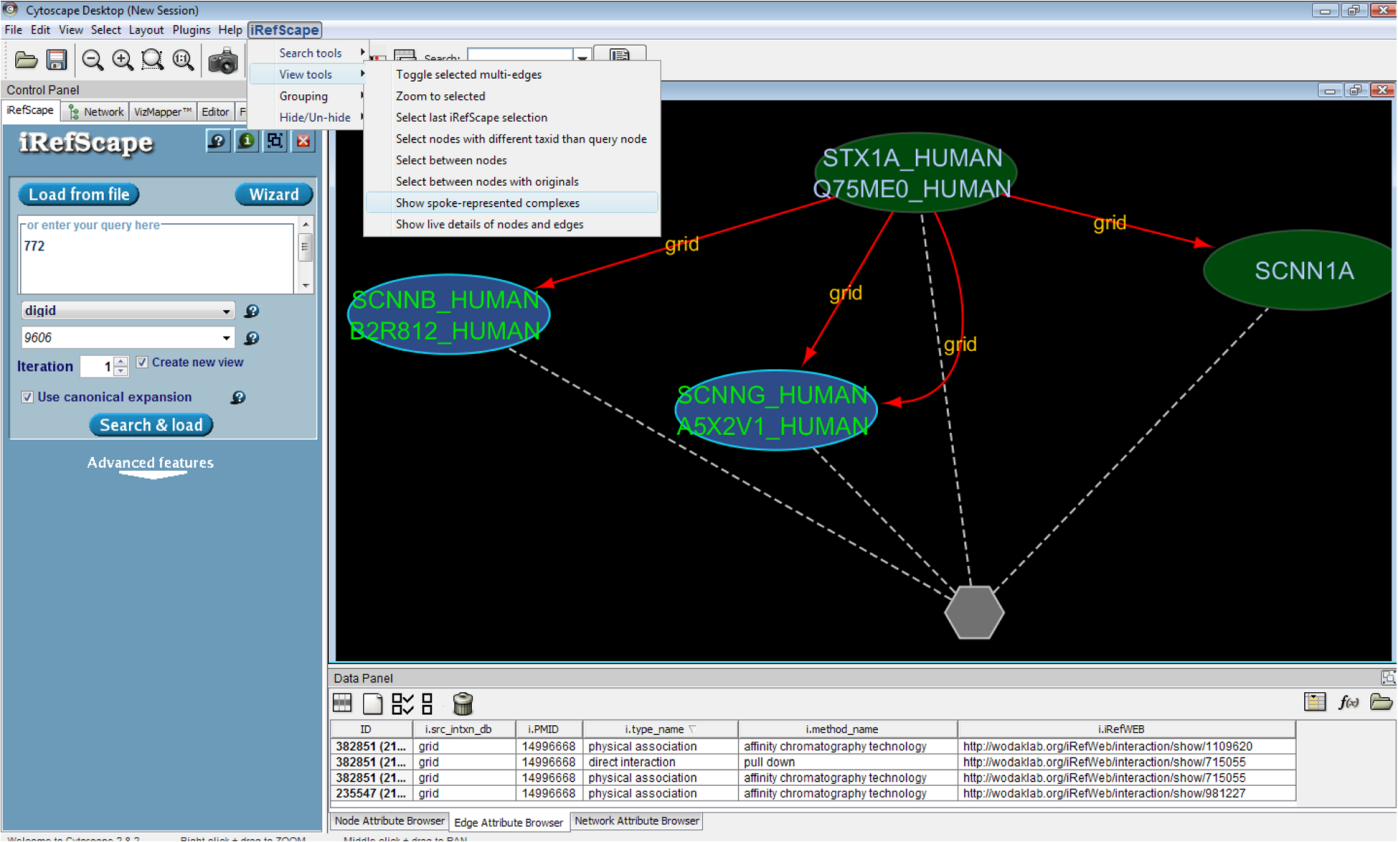
**Liddle syndrome.** Mutations in either SCNNB or SCNNG are associated with Liddle syndrome (disease group 772). Both are subunits of the heterotrimeric (alpha, beta, gamma) nonvoltage-gated, amiloride-sensitive, sodium channel. Both proteins were observed together with SCNN1A (the alpha subunit of the channel) as interactors with syntaxin 1A (STX1A). The original paper [32] contains evidence for a direct interaction between STX1A and the gamma subunit and for a complex that includes all four proteins using in-vitro translated components (Figure one in [32]). The complex is represented as four binary interactions in the BioGrid database. These interactions are identified as part of a potential spoke-represented complex by iRefScape (a grey hexagon appears after selecting View Tools/Show spoke-represented complexes from the iRefScape menu).

We reviewed papers for those diseases that had significant overlaps with the regenerated complexes (but not with n-ary or binary data). In most cases, our regenerated complexes were created from these papers because they all described a set of binary observations that all used the same method and the same bait protein. It was rare to find descriptions of bona fide complexes. Figure 4 illustrates one such exception – two subunits of a sodium channel complex held in common with the Liddle syndrome disease group.

Therefore, we concluded that while this method remains a formal possibility for finding overlaps between diseases and complexes in interaction data, it is not a reliable way of identifying complexes but a way to identify proteins that are usually related functionally. The majority of disease groups detected in overlaps using this method also had statistically significant overlaps with n-ary data and/or binary data (see below), therefore, the method is not critical for retrieving disease group overlaps (at least in this study).

The complex span (number of regenerated-complexes that match at least one subunit of a DiG) is a number between 0 and 1198 complexes. The significant matches (raw p-value < 0.05) are values between 1 and 1116, and the FDR-adjusted matches between 1 and 345. However, 95% of all DiGs with significant overlaps match only 11 or fewer regenerated complexes.

### Overlap between disease groups and binary data

Binary data is different to the complex data that we have analyzed so far, therefore we have reformulated our question as how probable it is to find a statistically significant number of binary interactions between the proteins belonging to a DiG. That is, in a network of 16272 proteins, there are 132,380,856 possible interactions but only 113,733 are documented. In order to find out how probable it is to find a certain number of interactions among the proteins belonging to a DiG, given this background, we performed a new hypergeometric test which uses a significant p-value of 0.0004 as a correction for the fact that the probability of randomly finding an interaction is dependent on the degree, as explained in Methods. Results for all DiGs are provided as Additional file 9.

**Figure 5:**
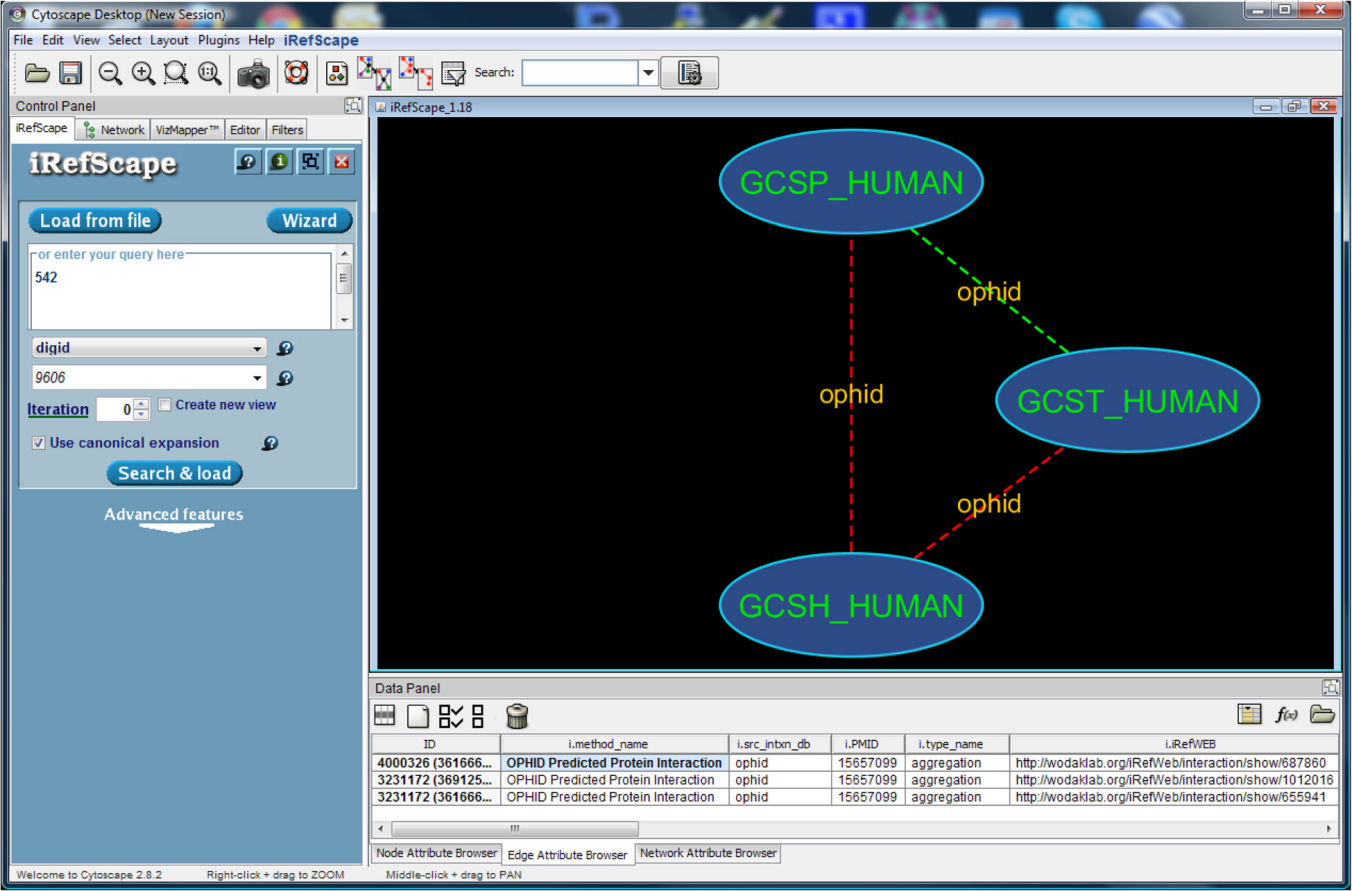
**Glycine encephalopathy.** Mutations in any one of three genes can cause Glycine encephalopathy (see MIM 605899 and disease group 542). All three potential pairwise interactions are found as predicted interactions in the OPHID database. No other database in iRefIndex includes these interactions. The three proteins are all part of the glycine decarboxylase complex; a loosely associated multienzyme complex consisting of four proteins that catalyzes the oxidative cleavage of glycine to carbon dioxide, ammonia, and a methylene group, in a multistep reaction. The fourth subunit (DLDH_HUMAN a.k.a. DLD or GCSL) has no interactions with any of the above three subunits in the iRefIndex. DLD is also a subunit of the branched-chain alpha-keto acid dehydrogenase complex (BCKD). Mutations in DLD or any other of the three catalytic subunits of this complex can lead to Maple Syrup Urine Disease (MIM 248600) – a disease with similar phenotype. This complex is not detected by any of the methods shown in this study since interactions between its subunits are not present in iRefIndex.

This table shows that 87 DiGs were found to be significantly enriched in binary interactions. Groups of proteins found to have association or over-representation can potentially have a biological meaning. Therefore, this result may reflect that there is a way of grouping proteins, related to the disease, for which we do not currently have a full explanation, although we can also hypothesize that they may correspond to unknown complexes, pathways, or other forms of functional organization. Again, a review of the literature underlying the interactions in each case is required before coming to a conclusion. These results simply provide a ranked list of those disease groups that are associated with an unexpectedly high number of binary interactions. The iRefScape plugin for Cytoscape can be used to explore specific disease groups (either by searching for the disease group or the associated list of proteins). Figure 5 shows one example involving Glycine encephalopathy.

### Combined list of disease group overlaps by interaction data type

Combining the lists of DiG overlaps related to n-ary, regenerated and binary data, we found a total number of 159 DiGs (out of 497 starting DiGs) that may be related to complexes or other forms of functional organization. The main contributors to this result were the regenerated n-ary and n-ary data sets followed by the binary data set.

We expect that our tables of significant matches will be useful to a more detailed study of the identity, similarity and nature of the matching complexes to a given DiG. However, this is not practical for DiGs that have overlaps with a large number of complexes. In these cases, a visual interface such as iRefScape can facilitate a review of the data – here similar complexes can more easily be identified and compared. However, a significant number of disease groups overlap with a manageable number of complexes and 66 out of the above 159 disease groups can be confidently related to one single protein complex (only one significant p-value after FDR adjustment).

**Figure 6:**
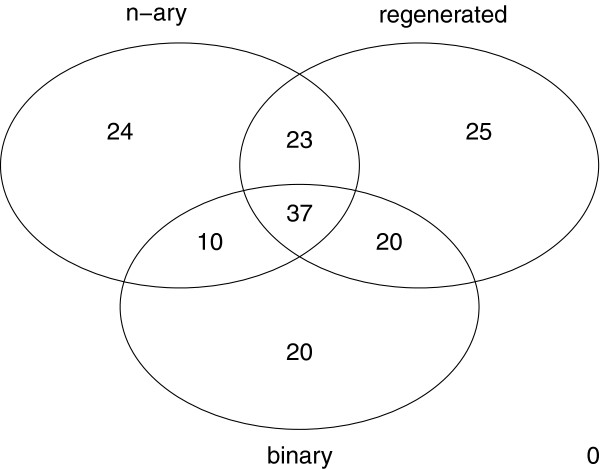
**Diagram of DiG overlaps with each of three different interaction data types.** A Venn Diagram shows that some DiGs correlate to a complex or complexes found at only one of the three protein information sources (24 DiGs when using n-ary data, 25 for regenerated complexes and 20 for binary interactions between the DiG proteins). At the same time, it can be seen that some DiGs are significantly similar to complexes found in more than one of the three protein interaction information sources.

These data are summarized in Figure 6 using a Venn diagram with the differences and coincidences between the DiG-interaction matches after using iRefIndex's n-ary data, regenerated data and binary data as sources. The diagram shows that some matches are found using two or three of the data sources indicating the presence of mutually supportive data for half of the disease groups with significant overlaps. A total of 37 DiGs have significant overlaps with all three data sets.

However, 24 DiGs only overlap with n-ary complexes, 25 overlap only with regenerated complexes, and 20 are only enriched for binary data. This suggests that the three data sources are complementary and should be used together in order to create a full picture. Tables 1, 2 and 3 are summary tables for the DiG overlaps found using only n-ary, regenerated or binary data respectively. Additional file 10 lists all disease groups and their best overlaps with each of the three data types. The reader can use these tables as a guide to diseases that have significant overlaps with interaction data. R code to retrieve reference information for each complex and DiG pair is provided in Additional file 1 (section 12).

**Table 1:** DiGs related only to complexes found in n-ary data

**DiG ID**	**DiG name**	**# Genes**	**Best match (icrigid)**	**Complex span**	**# significant p-values (raw)**	**# significant p-values (FDR)**
6	3-methylcrotonyl-coa carboxylase	2	1209480	9	9	2
65	Albinism	3	1209579	1	1	1
80	alport syndrome	3	781937	4	4	1
89	Amyloidosis	6	1221863	26	22	9
168	bethlem myopathy	3	1027975	5	5	1
192	Bradyopsia	2	969965	1	1	1
259	Ceroid	8	1232651	6	3	1
302	combined cellular and humoral immune defects	2	1220789	1	1	1
313	congenital disorder of glycosylation	23	725907	28	26	4
578	Hematuria	2	781937	4	4	1
595	hereditary hemorrhagic telangiectasia	2	618400	1	1	1
690	immune dysfunction	2	1122614	5	5	2
758	leigh syndrome	14	1211293	44	36	13
812	maple syrup urine disease	4	1225549	12	11	1
870	mitochondrial complex	16	1211293	28	24	5
975	omenn syndrome	3	1220789	1	1	1
998	Osteoporosis	5	869728	5	5	2
1108	Propionicacidemia	2	1209480	5	5	2
1266	stickler syndrome	3	878437	4	4	1
1341	tumoral calcinosis	4	682939	3	3	1
1345	ullrich congenital muscular dystrophy	3	1027975	5	5	1
1477	celiac disease	4	1220318	2	1	1
1512	intervertebral disc disease	2	893696	1	1	1
1520	Leprosy	4	651466	7	7	1

Summary of the 24 DiGs that can be related to protein complexes but can only be found in iRefIndex n-ary data: Mitochondrial complex deficiency (DiG ID = 870) is a DiG that groups 16 genes. At least one of the 16 genes in this DiG was present in 28 complexes present in iRefIndex n-ary data (complex span = 28) and 24 of them could be considered as significantly similar after a hypergeometric test with a p-value < 0.05. After adjusting the p-values for multiple testing using the FDR method, only 5 of those complexes could be considered significantly similar to the DiG, and therefore, their subunits related to the diseases involved in the DiG. The best match, among those 5, is the complex with the icrigid = 1211293, which corresponds to the complex in the CORUM database with interaction identifier = 15317750.

“3-methylcrotonyl-coa carboxylase” stands for “3-methylcrotonyl-coa carboxylase 1 deficiency” and “3-methylcrotonyl-coa carboxylase 2 deficiency”, while “Ceroid” stands for “Ceroid lipofuscinosis” and “Mitochondrial complex” stands for Mitochondrial complex I, II, III and IV deficiencies.

**Table 2:** DiGs related only to regenerated complexes

**DiG ID**	**DiG name**	**# Genes**	**Best match (database.pmid.exp_method.icrogid)**	**Complex span**	**# significant p-values (raw)**	**# significant p-values (FDR)**
63	alagille syndrome	2	MI:0463(grid).pubmed:10958687.MI:0004(affinity chromatography technology).1144108.MI:0463(grid).pubmed:10958687.MI:0096(pull down).1144108	4	4	1
190	Brachydactyly	7	MI:0463(grid).pubmed:9525338.MI:0096(pull down).248458	13	12	1
246	central hypoventilation syndrome	6	MI:0463(grid).pubmed:10829012.MI:0096(pull down).4168707	13	12	1
271	Chondrodysplasia	6	MI:0463(grid).pubmed:9525338.MI:0096(pull down).248458	13	13	1
290	cockayne syndrome	2	MI:0463(grid).pubmed:10944529.MI:0004(affinity chromatography technology).660979	11	11	2
363	Deafness	59	MI:0463(grid).pubmed:12485990.MI:0096(pull down).1981308	134	56	1
424	endometrial carcinoma	5	MI:0463(grid).pubmed:8942985.MI:0096(pull down).813561.MI:0463(grid).pubmed:10029069.MI:0096(pull down).813561.MI:0463(grid).pubmed:9774676.MI:0096(pull down).813561.MI:0469(intact).pubmed:9774676.MI:0096(pull down).813561	105	103	5
451	Exostoses	2	MI:0463(grid).pubmed:17353931.MI:0004(affinity chromatography technology).3748087.MI:0469(intact).pubmed:17353931.MI:0006(anti bait coip).3748087	13	13	1
512	gastric cancer	11	MI:0469(intact).pubmed:19411071.MI:0006(anti bait coip).3231405	194	190	2
687	Ichthyosis	13	MI:0469(intact).pubmed:17373842.MI:0006(anti bait coip).1386965	25	21	1
714	jackson-weiss syndrome	2	MI:0463(grid).pubmed:20388777.MI:0004(affinity chromatography technology).3027803	16	16	1
730	Keratosis	6	MI:0463(grid).pubmed:11790773.MI:0004(affinity chromatography technology).2791010	65	60	5
772	liddle syndrome	2	MI:0463(grid).pubmed:14996668.MI:0004(affinity chromatography technology).382851	14	14	8
829	medullary thyroid carcinoma	2	MI:0463(grid).pubmed:8183561.MI:0004(affinity chromatography technology).1871880	26	26	1
904	Mycobacterium	10	MI:0463(grid).pubmed:10848598.MI:0004(affinity chromatography technology).1004542	45	42	2
933	nephrotic syndrome	4	MI:0463(grid).pubmed:11733557.MI:0004(affinity chromatography technology).3453124.MI:0463(grid).pubmed:11733557.MI:0096(pull down).3453124	8	8	1
959	noonan-like/multiple giant cell lesion syndrome	2	MI:0463(grid).pubmed:9344843.MI:0004(affinity chromatography technology).5369795	100	100	1
999	Osteosarcoma	2	MI:0463(grid).pubmed:12242661.MI:0004(affinity chromatography technology).3633225	340	340	318
1049	pfeiffer syndrome	2	MI:0463(grid).pubmed:20388777.MI:0004(affinity chromatography technology).3027803	16	16	1
1051	pheochromocytoma	6	MI:0463(grid).pubmed:10829012.MI:0096(pull down).4168707	115	109	1
1071	pituitary hormone	5	MI:0463(grid).pubmed:10788441.MI:0096(pull down).3747010	9	9	1
1165	rhabdomyosarcoma	4	MI:0463(grid).pubmed:17662948.MI:0004(affinity chromatography technology).2068280	30	30	1
1233	Sitosterolemia	2	MI:0471(mint).pubmed:16870176.MI:0007(anti tag coip).3242301	2	2	1
1260	squamous cell carcinoma	3	MI:0463(grid).pubmed:15659383.MI:0401(biochemical).4258047	52	52	12
1574	Tuberculosis	3	MI:0463(grid).pubmed:7673114.MI:0096(pull down).4202157	9	9	1

Summary of the 25 DiGs that are significantly similar to protein complexes but can only be found in iRefIndex regenerated complex data: Ichthyosis (DiG ID = 687) is a DiG that groups 13 genes. At least one of the 13 genes in this DiG was present in 25 complexes present in iRefIndex regenerated n-ary data (complex span = 25) but only 21 of them could be considered as significantly similar after a hypergeometric test with a p-value < 0.05. After adjusting the p-values for multiple testing using the FDR method, only 1 of those complexes could be considered significantly similar to the DiG.

**Table 3:** DiGs enriched only in binary interactions

**DiG ID**	**DiG name**	**# Genes**	**# binary interactions in DiG**	**p-value**
156	basal cell carcinoma	4	2	1.1e-05
263	charcot-marie-tooth disease	26	4	2.0e-4
305	Immunodeficiency	12	4	3.8e-07
310	retinal dystrophy	22	4	5.4e-05
379	diabetes mellitus	44	12	7.7e-11
538	Glutaricaciduria	4	3	1.3e-08
542	glycine encephalopathy	3	3	6.3e-10
543	glycogen storage disease	19	6	1.1e-08
581	Hemochromatosis	5	2	3.3e-05
626	Hypercholesterolemia	9	3	4.4e-06
628	Hyperekplexia	5	2	3.3e-05
644	Hyperphenylalaninemia	4	2	1.1e-05
780	Lissencephaly	5	2	3.3e-05
850	Methemoglobinemia	4	2	1.1e-05
996	Osteopetrosis	8	2	2.7e-4
1081	polycystic kidney	4	2	1.1e-05
1092	Porphyria	6	5	1.4e-12
1153	retinitis pigmentosa	43	8	1.6e-06
1300	Thrombocythemia	3	2	2.2e-06
1536	myocardial infarction	13	4	7.4e-07

Summary of the 20 DiGs that are enriched in iRefIndex binary interactions and can only be found using this method: Glycine encephalopathy (DiG ID = 542) is a DiG that groups 3 genes. There are 3 possible interactions between 3 genes. Finding all of them in the PIN is a very unlikely event (hypergeometric p-value close to zero) and, therefore, the existence of a functional group of proteins related to the disease can be hypothesized.

“immunodeficiency” stands for immunodeficiencies due to defects in CD3, MAPBP-interacting protein, with hyper IgM and X-linked.

### Analysis per database and study type

We examined the list of distinct n-ary complexes that had the best significant overlap with each disease group in order to investigate the primary databases in iRefIndex from which matching complexes were retrieved. There are 79 distinct complexes overlapping with 94 disease groups. Table 4 shows that these are described in seven different primary databases (IntAct, HPRD, CORUM, DIP, InnateDB, MINT and BIND), which high-lights the importance of interaction database integration as a requisite to explore relationships to diseases. We note that 61 out of the 79 best matching complexes are derived from low-throughput studies (as indicated by an lpr score smaller than 22: see Methods), which indicates that they preferentially come from low throughput experiments and that the results are not dependent on high-throughput studies.

**Table 4:** Sources of significant n-ary data

**Database(s)**	**Number of best matches to DiGs**
IntAct	24
CORUM	16
HPRD	13
DIP	6
InnateDB	5
DIP & IntAct	5
Mint	4
BIND & CORUM	2
DIP, MINT & InnateDB	1
BIND	1
CORUM, IntAct & HPRD	1
CORUM & IntAct	1

Number of best matches to DiGs per source database. 7 different databases contribute with complexes that match at least one DiG. In 87% of the cases the complex belongs to only one database. The other 13% can be found in 2 or 3 databases at the same time. The table shows how using only a subset of databases instead of a consolidated data set may lead to incomplete results regarding DiG matches.

We also examined the list of distinct regenerated complexes that had the best significant overlaps with each disease group. There were 89 distinct complexes overlapping with 105 disease groups. Table 5 shows that the 89 best matching regenerated complexes are derived almost entirely from BioGRID [33] while only a few come from IntAct and MINT. This is expected since BioGRID represents n-ary data using a spoke model (the intended target of regenerated complexes).

**Table 5:** Sources of significant regenerated data

**Database(s)**	**Number of best matches to DiGs**
BioGRID	73
IntAct	8
Mint	3
BioGRID & IntAct	3
BioGRID & Mint	1
IntAct & Mint	1

Number of best matches to DiGs per source database. 3 different databases contribute with regenerated complexes that happen to match at least one DiG. BioGRID is the main contributor to this group of complexes. The table shows that the process of regenerating potentially spoke-represented complexes may be important to detect information on protein groups matching DiGs.

For binary interactions, 12 out of 13 databases in the “iRefIndex” provide human binary data (these are all the above-mentioned databases plus OPHID [34], MPPI [35] and MatrixDB [36]). OPHID is a source of predicted interactions in human based on orthologous transfer from observations in other experimental organisms. OPHID did not have overlaps with diseases in the n-ary (complex) data set since predicted interactions are based solely on binary interaction records. Given that 94 disease groups significantly overlapped with the n-ary records that are present for human, we speculate that orthologous transfer of n-ary data from other experimental organisms to human could provide a useful source of information.

### Overlap between disease groups and pathways

As a complementary analysis, we decided to evaluate the overlap between our DiGs and a pathway database. We selected the KEGG database [37], although the analysis could be extended to others.

**Figure 7:**
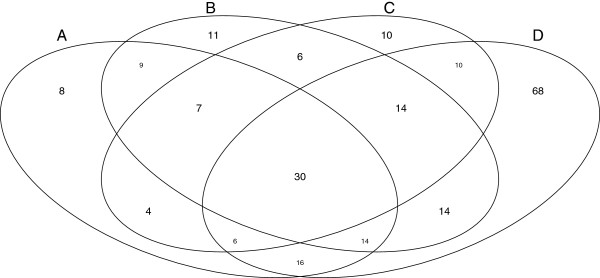
**Diagram of DiG overlaps with three different interaction data types and KEGG data.** A Venn diagram of the number of disease groups that have significant overlaps with each of the three interaction data types and the KEGG pathway database. **A**. n-ary data. **B**. regenerated complex data. **C**. binary data. **D**. KEGG data. Diseases lacking significant overlaps with KEGG are evenly spread among the three interaction types.

Additional file 11 contains detailed results. In summary, KEGG pathways match 172 DiGs (p-value < 0.05, FDR adjusted): 104 matches already detected by interaction sources and 68 new matches. Therefore, KEGG lacked significant overlaps with only 55 disease groups that had overlaps in the iRefIndex data. Figure 7 shows a detailed Venn diagram of overlaps between disease groups and all three interaction data types plus pathway data.

These results suggest that pathway and interaction data sources may be complementary although further examination of individual cases will be required to confirm this. This work is non-trivial and we suggest that the disjoint lists of diseases found by either KEGG or by interaction databases alone would make a good starting point for a collaborative curation project to investigate further the disease coverage of these data sets. In the meantime, there is good reason to consult both interaction and pathway data when performing disease-related analyses [14].

### Change of results over time

In order to see how conclusions change with time, we have compared all previous analyses between the iRefIndex versions 8.0 (January 19^th^, 2011) and 9.0 (October 18^th^, 2011). Table 6 shows that there is an increase in interaction records, both binary and complex, and disease genes with interaction information, from one version to the next. The direct consequence of this is that there is also an increase in n-ary and regenerated groups, as well as binary-enriched DiGs, and an increase in matches between DiGs and all three sources. Moreover, it is important to highlight that not only the number of DiG-complex matches grows but also the number of matches detected with only one of the data sources (at least for n-ary and regenerated data), indicating that using different sources has become more important with time. Table 6 also shows that more databases contribute with significant information to the analysis (InnateDB was not present in iRefIndex 8.0), while the number of databases with spoke-represented complexes in need of being regenerated remains the same (BioGRID, IntAct, MINT). Finally, 9 of the matches detected using KEGG but not interaction data from iRefIndex 8.0 were found using iRefIndex 9.0. The methods and scripts developed here will provide us with the means to monitor how these trends progress over time.

**Table 6:** Comparison of results using iRefIndex version 8.0 and iRefIndex 9.0

**Variable**	**iRefIndex 8.0**	**iRefIndex 9.0**
# Human-Human Interaction records	319447	361059
# Genes in Morbid Map with PI info in iRefIndex	1685	1719
# Genes in Morbid Map without PI info in iRefIndex	256	222
# DiGs with non-translatable genes	166	156
# n-ary groups	4827	5677
# regenerated groups	7830	9947
# binary nodes in PIN	15597	16272
# binary edges in PIN	98853	113733
# significant matches DiG-nary	81	94
# significant matches DiG-regenerated	96	105
# binary-enriched DiGs	84	87
# all significant DiGs	220	227
# DiG matching only nary data	16	24
# DiG matching only regenerated data	22	25
# DiG matching only binary data	21	20
# databases with nary data matching DiGs	6	7
# databases with regenerated data matching DiGs	3	3
# nary groups with lpr < 22 (low-throughput)	53	61
# Matches found using KEGG and not found using iRefIndex	77	68

The first 8 rows show how knowledge about the human interactome grows from one database release to the next; that is, the growth of the number of interaction records (319447 to 361059), the number of genes associated to disease in OMIM having interaction information in iRefIndex (1685 to 1719), the number of complexes coming from n-ary (4827 to 5677) and regenerated (7830 to 9947) data, and the number of binary nodes and edges in the PIN. This leads to an increase in the number of matches between DiGs and each of the three data sources (nary, regenerated, binary) and, in total, an increase from 220 to 227 matching DiGs from one release to the following one. The number of DiGs matching only one type of data also grows, as well as the number of databases with n-ary data matching DiGs.

## Discussion

Our overview of the relationship between diseases and protein interaction data indicates that roughly half of our disease groups could potentially be explained in terms of groups of proteins, which may be complexes, spoke-represented complexes, groups of binary interactions or pathways. These matches can occur between a disease group and either one or many protein groups (complex span). The situation as of release 9.0 of the iRefIndex is detailed in the different tables and supplementary tables.

In summary, 227 DiGs (out of 497 multigenic DiGs) show significant similarity to a number of n-ary complexes (94), regenerated complexes (105), binary-groups (87) or pathways (172) and the number of matches per DiG ranges between 1 and 345, numbers that seem to increase as databases grow.

We have shown that disease group overlaps can be found within three types of interaction data: n-ary, spoke-represented complexes and binary. These sources are complementary and should be examined separately when searching for overlaps. It is particularly interesting that a large number of distinct overlaps were found with spoke-represented data: a data set that would not normally be amenable to overlap detection unless the analyst specifically attempted to detect binary data that was potentially describing a protein complex. However, we found that this data set by itself was unable to reliably identify bona fide complexes when we examined the source papers even though it was identifying a functionally related group of proteins.

One goal of this work was to assess how important data integration is to this type of study. Since interaction databases are so redundant and since there are already databases that have specialized in capturing human-specific information and even complex data, one might conclude that not all data resources are really required. However, we found that interaction data and pathway data appear to be complementary and that overlaps with all three types of interactions included records from several databases. Therefore integration of data sources is an important issue if analysts are to detect all significant overlaps with a disease. The quality and usefulness of these overlaps from individual databases is beyond the scope of this survey. This should be addressed in future. Here, we have found sufficient evidence to show that no single database has systematically subsumed all significant disease group overlaps and that all diseases have not been thoroughly covered by the collective set of databases examined here. We would argue that this could be addressed by future collaborative efforts between these databases.

It is important to qualify these results and place them in the context of future studies. First, our method of creating disease groups is a fairly conservative and simple string-matching technique that could be replaced by more sophisticated methods [7] or disease-phenotype classifications. Further, examination of these statistically significant overlaps is required to assess how often these groups truly represent relevant complexes or pathways that can offer an explanatory basis for a disease group. The accompanying tables, scripts and the iRefScape plugin provide the means to direct, facilitate and track further study and curation efforts. These could be directed towards the significant overlaps found here and also towards the disease groups for which we did not find overlaps in interaction data or pathway data.

In the meantime, the data presented in this survey and the disease group search functionality in the iRefScape plugin can act as a guide towards searching for disease-relevant interaction data across a number of databases.

## Conclusions

This paper has examined the current relationship between diseases and protein interaction data. However, these results will change over time and, therefore, we prefer to place emphasis on the importance of data integration, the variety of data types that need to be taken into account and the tools we have developed for these analyses, which can allow future researchers to access updated results.

The predictions obtained from our results could be used as a feedback to interaction, complex and disease databases. iRefIndex includes no disease annotation, due to the fact that complex records in interaction databases rarely contain references to diseases or to OMIM. The work presented here could be used to systematically review and put in place cross-references between OMIM and interaction databases.

## Methods

All analyses were performed using R 2.14.1 and iRefR v. 0.94. Additional R libraries used in this paper include “limma”, “gplots”, “moments”, “org.Hs.eg.db” and “igraph (version 0.5.5)”. The “iRefR”, “gplots” and “moments” packages can be installed from CRAN [38], while “limma” and “org.Hs.eg.db” can be installed from Bioconductor [39]. All code necessary to replicate the R analyses in this paper (except the construction of DiGs) is provided as Additional file 1. The Cytoscape graphs were generated using Cytoscape 2.8.2 and iRefScape 1.18 using version 9.0 of iRefIndex (see http://irefindex.uio.no).

### Construction of consolidated protein interaction data set

Protein interaction data was consolidated from 13 different databases using the iRefIndex procedure described previously [11,19]. Each node in the resulting PIN represents a distinct sequence from a distinct organism. Further, groups of related proteins (for example, splice isoforms) are represented by a single representative from each group (canonical representation). As a result, each Entrez GeneID in a disease group will map (at most) to a single node in this PIN.

The final network (iRefIndex release 9.0 – October, 2011) describes (after consolidation) 401,140 distinct human protein-protein interactions. A subset of interactions where both interactors were human proteins was used in this study and contained 361,059 distinct interactions. The corresponding PSI-MITAB file used in this study is available at ftp://ftp.no.embnet.org/irefindex/data/current/psimi_tab/MITAB2.6/ or can be downloaded using iRefR [14] or searched and viewed using iRefScape [19].

### An explanation of interaction data types and their representation

**Figure 8:**
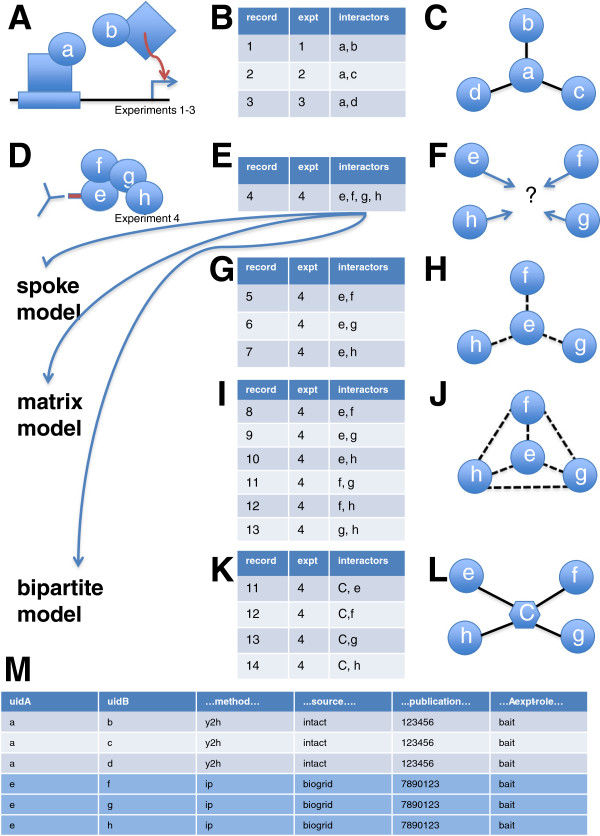
**An explanation of interaction data types and their representation.** Experiments that can be used to detect interactions may produce *binary* data (panels **A**-**C**) or *n-ary* data (panels **D**-**F**). N-ary data is commonly represented using one of three different models: the spoke model (panels **G**-**H**), the matrix model (panels **I**-**J**) or the bipartite model (panels **K**-**L**). Some n-ary data is represented using a spoke model and an attempt can be made to detect these records in MITAB files and reconstruct the list of component proteins in a potential complex (panel **M**). These lists are referred to as *regenerated complexes* in this paper. The full explanation of this Figure is provided in the Methods section.

Figure 8 shows a detailed explanation of the different interaction data types and their representations used in this paper. This figure describes binary data, n-ary data and regenerated complexes as well as spoke, matrix and bipartite models for representing n-ary data.

A. **A binary type experiment.** The yeast two-hybrid method is a typical binary type method. Two fusion proteins are created: a transcription factor DNA-binding domain fused to a bait protein (a) and a transcription activation domain fused to a prey protein (b). An interaction between a and b restores transcriptional activation (red arrow) of a reporter gene that can be detected in an *in vivo* assay (experiments 1–3).

B. **Results of a binary experiment.** The results of three different experiments (experiments 1–3, each using bait protein a and one bait protein (b, c or d)) are recorded in three different interaction records and provide evidence for interactions between a and b, a and c and a and d. Other types of experiments can produce binary data. What binary type methods have in common is that the output of each experiment produces a record with only two proteins. Depending on the experiment type, the results may constitute evidence for a direct interaction (e.g. yeast 2-hybrid) or an indirect interaction (e.g. synthetic lethal screen).

C. **Graphical representation of a binary experiment.** The results of experiments 1–3 can be represented using a graph. Proteins a, b, c and d are the nodes and edges between nodes represent the fact that experimental evidence exists supporting an interaction between those two proteins.

D. **An n-ary type experiment.** An immunoprecipitation pull-down assay is a typical n-ary type method. A bait protein (e) is fused to an epitope and expressed *in vivo*. Cell lysate is immunoprecipitated using an antibody specific to the epitope tag (red) on protein e. Proteins binding directly (or indirectly) to e are captured as part of the immunoprecipitate and detected using some method such as mass spectrometry.

E. **Results of an n-ary experiment.** The results of the immunoprecipitation and detection (experiment 4) are captured in a single interaction record that lists all of the observed proteins (e, f, g, h) and notes the protein used as bait (e). Other types of experiments can produce n-ary data. What n-ary type methods have in common is that the output of each experiment can produce a record with three or more proteins. N-ary results may be represented as a list of protein interactors using the PSI-MI XML format (see http://www.psidev.info/node/60). However, n-ary results cannot be represented in a single MITAB formatted record because each record (in MITAB format) can list no more that two interaction participants (panel M). In order to represent these data in MITAB format, they must first be transformed into a binary-like format using one of three methods: panels (G-K).

F. **Graphical representation of an n-ary experiment.** The results of an n-ary type experiment cannot be rendered as a graph like the one in panel C. The experimental observations show that the proteins in the list are somehow associated but contain no information about interactions between any given pair of proteins. However, n-ary data is quite commonly transformed using one of three methods (panels G, I, K) such that they can be represented using a graph and combined with binary data like that shown in panel C.

G. **Spoke representation of n-ary data.** Interaction records are created for each protein (f, g, h) that was observed to be associated with the bait protein (e). Protein e is called the *hub* and each of its *interactions* with the observed prey proteins is called a *spoke*. In n-ary experimental systems that do not use a bait protein (e.g. x-ray crystallography of a multi-subunit complex), one of the observed proteins will be arbitrarily chosen to represent the hub in the spoke model. Spokes do not constitute evidence for an interaction; they serve to group together proteins in a series of binary records that were all associated in one experimental result. Each of these records will list the same study, the same n-ary method and the same bait protein (if applicable). Therefore, in a MITAB record (see panel M below), the fact that the results of an n-ary experiment are being represented with a spoke model is implicit – it is left to the user to notice if an n-ary type method is being used and that there are other records (in the same MITAB file) that share a common experimental reference and bait. With the recent introduction of MITAB version 2.7, a new column (called *expansion*), explicitly states if the record is part of a spoke model. See http://code.google.com/p/psicquic/wiki/MITAB27Format.

H. **Graphical representation of spoke data.** The data from panel E can be represented in a graphical format. Note that the topology of graphs in panels C and H are identical even though the meaning is completely different; the presence or absence of an edge between protein nodes does not constitute evidence for or against an interaction.

I. **Matrix representation of n-ary data.** Interaction records are generated for all possible pairwise combinations of proteins observed in experiment 4. This representation is less common than the spoke model. Like the spoke model, this representation serves to group together members of a list of proteins that are experimentally observed to be associated.

J. **Graphical representation of matrix data.** The data from panel I can be represented in a graphical format. The same caveats apply as in panel H (except that there will be no missing edges).

K. **Bi-partite representation of n-ary data.** An artificial node is created (C) to represent the idea of a complex. Interaction records are made for each protein observed in experiment 4 and this complex node. This representation is the least common. It is used by iRefIndex to represent n-ary data in its MITAB file distribution – such records cannot be mistaken for binary records because they each list only one protein. Source database records that are of the form shown in panel E (a list of proteins) will be represented in the iRefindex MITAB using this model. However, source database records that were distributed as a spoke model (as in panel G) will not be represented this way by iRefIndex. In these cases, we must use a heuristic algorithm to try to detect those sets of binary records that are likely to be spoke representations of n-ary data – an n-ary record can be inferred from this search (see panel M). In this paper, we call these inferred n-ary records *regenerated complexes*.

L. **Graphical representation of bi-partite data.** The representation shown in panel K can be rendered in a graphical format. A circular node represents a protein. A hexagonal node represents a complex node. An edge between a protein and a complex node represents membership of the protein in that complex. The graph uses two node types and is therefore referred to as a bi-partite graph.

M. **MITAB format.** A MITAB file is a tab-delimited text file where each row represents one interaction record. This panel shows selected fields of a MITAB file for experimental results in panel B (results of a binary experiment) and panel G (spoke representation of results from an n-ary experiment). The first three rows constitute evidence for interactions between a and b, a and c and a and d. The last three rows are *potentially* spoke representations of a single immunoprecipitation experiment where e was observed to be associated with proteins f, g, and h. We can infer that *this set of records* supports the idea of a complex between e, f, g and h because 1) they all share a method that is known to potentially generate n-ary (immunoprecipitation), 2) they all come from the same source database (BioGrid), 3) they all refer to the same publication and they all involve the same bait protein (experimental role of A is bait in all three cases). In this paper we therefore create a regenerated complex composed of the list of proteins e,f,g,h. This list is amenable to the hypergeometric test. However, it is also possible that these three records are all descriptions of experimental results from three separate experiments in the same paper – in which case there is no evidence of an association that involves these four proteins at the same time. In most cases, it is impossible to distinguish between these two cases by just looking at the XML or MITAB version of the interaction record and a re-reading of the paper is necessary to confirm the legitimacy of the regenerated complex.

### Construction of disease groups

The OMIM Morbid Map (June 14^th^, 2010) was obtained from the OMIM FTP site [20]. Each entry in this table describes a relationship between some OMIM disease entry and some gene from Entrez Gene. Each entry contains 4 pipe-delimited columns that list (1) the disease title, OMIM identifier referring to the disease and an evidence code, (2) the official gene symbol and its synonyms for the gene that is related to this disease, (3) the OMIM identifier referring to this disease-gene relationship and (4) locus information for the associated gene. We restricted our analysis to those entries with evidence code 3 meaning that evidence for an association between the disease phenotype and the gene was obtained by mapping both a mutation associated with the disease to the wild-type gene and by mapping the disease phenotype itself to the gene [40]. The Morbid Map was pre-processed to assign integer Entrez GeneIDs to each Morbid Map entry. Gene symbols from column 2 were used to search for the corresponding Symbol in EntrezGene’s gene_info table and restricting search results to taxon identifier 9606 (human) and ensuring that the corresponding map location information matched what was given in the Morbid Map entry. In those cases where no hit was found, the search was broadened to include matches to Synonyms and LocusTag entries in the gene_info table.

Groups of disease-gene associations were constructed using disease titles (first column in the Morbid Map). In some cases, multiple entries have identical titles; these were assigned to the same Disease Group Identifier (DiG ID). However, titles were not always exactly the same and varied in the detail given. These variations often describe disease sub-types or are a result of simple naming inconsistency. Regular expression rules were used to group identical or similar Morbid Map entries under a common DiG ID. To initiate the text search, titles in column 1 were stripped of everything following the first comma (in the absence of a comma, the disease tag and OMIM identifier were removed) or everything following keywords “due to” or “with” that tend to specify subtypes of a main phenotype. Before the search for matching titles, white spaces, stand-alone digits and roman numerals were also removed. The resulting search strings were anchored at the beginning to select for partial title hits. Two kinds of opening brackets (“{“,”[“) were allowed to occur at the beginning - these brackets are used by OMIM as a special symbol to indicate susceptibility to certain conditions. A Structured Query Langage (SQL) regular expression search was used to form an initial group of hits. Every partial hit was examined. To be accepted, the match had to either end at the word boundary or with punctuation (“,”,”-”,”/”,”(“) or to continue with allowed suffixes (presently “s”, “tous”, “tosis” to accommodate matches of, for example, adenomatous and adenomatosis). Testing for word boundaries was necessary to exclude false matches that result from partial word matching. All approved matches were assigned the same integer DiG ID.

### Regenerated complexes

The process of regenerating spoke-represented complexes has been described in our previous work [14]. We used “iRefR” to regenerate complexes, following a canonical representation of proteins and the default list of experimental methods, that is, the methods with the following MI-ontology terms: MI:0004, MI:0006, MI:0007, MI:0019, MI:0027, MI:0028, MI:0029, MI:0059, MI:0061, MI:0071, MI:0096, MI:0114, MI:0226, MI:0227, MI:0401, MI:0437, MI:0676, MI:0858 and MI:0963 (see [41] under “interaction detection method”). As a result, 9947 human complexes were included in the analysis.

This reconstruction algorithm doesn't guarantee that we are identifying biologically functional complexes, but it guarantees that these proteins always co-purify and were reported in the same paper and database, which differs from other methods of generation of complexes from binary data such as clustering methods.

### Translating Entrez gene IDs to proteins

Entrez GeneIDs in DiGs were mapped to canonical group identifiers (icrogid) using “iRefR”, which, in turn, uses the information from iRefIndex version 9.0. This method ensures that each gene maps to, at most, one protein in the iRefIndex data set.

156 DiGs had genes with no corresponding protein (icrogid). These genes were included in all analyses as non-mappable entities, meaning that they could not match any protein in a complex but they are still a member of the disease group for statistical purposes.

### Assessing overlap between disease groups and complexes

Following Goldberg *et al*. [31], we used four similarity metrics: the Jaccard, Meet/Min, Geometric and Hypergeometric indexes, and we observed that hypergeometric indices gave the best results. We implemented two types of hypergeometric test:

In the first type, the population corresponds to the total number of distinct proteins in DiGs plus complexes (or pathways), the success population corresponds to the proteins in complexes (or pathways), the sample corresponds to each DiG, and the success sample to the intersection between each DiG and complex (or pathway).

In this case, the null hypothesis that the two groups (DiG and protein group) were independent was rejected if the corrected p-value was ≤ 0.05, i.e., a p-value smaller than a cutoff of 0.05 indicated a match. The resulting p-values were adjusted for multiple hypothesis testing (each complex or pathway considered as one different test) using the Bonferroni, False Discovery Rate and Benjamini-Yekutieli methods, using “p.adjust” in R. Bonferroni was the most conservative correction and FDR the less conservative, therefore we used the FDR adjusted p-value every time we spoke of the best match or the number of significant matches.

The second type of test was developed for binary interaction data. Here, the population corresponds to all possible binary interactions between proteins in the binary graph, the success population corresponds to the actual number of binary interactions reported, the sample corresponds to the size of all possible interactions among the proteins in a DiG, and the success sample to the actual number of binary interactions between the proteins of that DiG.

This type of test has the limitation that it considers the probability of finding an interaction for a node is equally probable for all nodes; however, the fact is that it is easier to find an interaction for a node with a high degree than for a node with a low degree. In order to correct for this, we performed 10,000 Monte-Carlo simulations for each disease group, selecting proteins of similar degree to the members of the real DiG for each simulation and counting the number of interactions in the simulated subgraph. The obtained results were equivalent to those of the uncorrected hypergeometric test using a cutoff of a p-value < 0.0004 and, therefore, we used the hypergeomtric test with the smaller cutoff as our final method instead of the more time-consuming simulations.

## Competing interests

The authors declare that they have no competing interests.

## Authors’ contributions

AM performed all analyses in this paper and wrote all R code for those analyses. KM developed the Disease Groups and performed the Monte-Carlo simulations used in the binary-enrichment analysis. IMD supervised the project and provided the Cytoscape examples. AM and IMD wrote this paper. All authors read and approved the final manuscript.

## Supplementary Material

Additional file 1 **This is a plain text file that contains R code to reproduce all R analyses in the paper.** See http://www.r-project.org/. Click here for file

Additional file 2 Distribution of number of genes per disease group (DiG). Click here for file

Additional file 3 Mapping of OMIM titles to disease groups and Entrez Gene identifiers. Click here for file

Additional file 4 Summary of disease groups in terms of both genes and proteins. Click here for file

Additional file 5 **The file can be opened in Cytoscape (**http://cytoscape.org**) to reproduce Figure **1** and explore disease groups and their overlaps.** Click here for file

Additional file 6 **Overlaps between disease groups and n-ary records showing best overlaps (icrigid) and number of overlaps (complex span) before and after correction for multiple hypothesis testing.** Additional information on n-ary records with the best overlap with a disease group can be found at http://wodaklab.org/iRefWeb/interaction/show/xxx where xxx is the icrigid ( for example, http://wodaklab.org/iRefWeb/interaction/show/705064). Click here for file

Additional file 7 **The file can be opened in Cytoscape (**http://cytoscape.org**) to reproduce Figure **3** and explore disease groups and their overlaps with n-ary data in iRefIndex.** Click here for file

Additional file 8 **Overlaps between disease groups and regenerated complex data showing best overlaps and number of overlaps (complex span) before and after correction for multiple hypothesis testing.** Example regenerated complexes have regular names that can be used to retrieve the binary interactions that make up the regenerated complex. For example: in the name “MI:0463(grid).pubmed:10722728.MI:0004 (affinity chromatography technology).10724593”, the BioGrid database has curated interactions from the paper with PubMed Identifier 10722728 where an affinity chromatography method was used to identify interactors of a common bait (icrogid: 10724593). Click here for file

Additional file 9 **Significance of overlaps between disease groups and binary data was calculated as described in the text.** Number of interaction edges for each disease group is listed. Only those disease group overlaps with raw p-values less than 0.0004 are considered statistically significant. Click here for file

Additional file 10 **Each disease group is listed along with its number of genes, Entrez Gene IDs, and best overlaps with n-ary data and regenerated data and most significant raw p-value for binary data enrichment.** Additional information on best overlapping n-ary record or regenerated complex can be found using the provided identifier as described in AF6 and AF8. Binary data corresponding to a disease group can be found using the iRefScape plugin for Cytoscape using the provided DiG ID or list of Gene IDs. Click here for file

Additional file 11 **Each disease group is listed along with the number of significant overlaps with KEGG pathway records before and after correction for multiple hypothesis testing.** The KEGG entry identifier for the best overlapping pathway record is provided in column 3. For example 5200 is record hsa05200. Click here for file

## References

[B1] International Classification of Diseases (ICD) http://www.who.int/classifications/icd/en/

[B2] Cornet R de Keizer N Forty years of SNOMED: a literature review BMC medical informatics and decision making 2008 8 Suppl 1 S2 19007439 10.1186/1472-6947-8-S1-S2 PMC2582789

[B3] McKusick VA Mendelian Inheritance in Man and its online version, OMIM American journal of human genetics 2007 80 4 588 604 17357067 10.1086/514346 PMC1852721

[B4] Becker KG Barnes KC Bright TJ Wang SA The genetic association database Nature genetics 2004 36 5 431 432 15118671 10.1038/ng0504-431

[B5] Feldman I Rzhetsky A Vitkup D Network properties of genes harboring inherited disease mutations Proceedings of the National Academy of Sciences of the United States of America 2008 105 11 4323 4328 18326631 10.1073/pnas.0701722105 PMC2393821

[B6] Goh KI Cusick ME Valle D Childs B Vidal M Barabasi AL The human disease network Proceedings of the National Academy of Sciences of the United States of America 2007 104 21 8685 8690 17502601 10.1073/pnas.0701361104 PMC1885563

[B7] Lage K Karlberg EO Storling ZM Olason PI Pedersen AG Rigina O Hinsby AM Tumer Z Pociot F Tommerup N A human phenome-interactome network of protein complexes implicated in genetic disorders Nature biotechnology 2007 25 3 309 316 10.1038/nbt1295 17344885

[B8] Dietmann S Georgii E Antonov A Tsuda K Mewes HW The DICS repository: module-assisted analysis of disease-related gene lists Bioinformatics 2009 25 6 830 831 19176557 10.1093/bioinformatics/btp055

[B9] da Huang W Sherman BT Lempicki RA Systematic and integrative analysis of large gene lists using DAVID bioinformatics resources Nature protocols 2009 4 1 44 57 10.1038/nprot.2008.211 19131956

[B10] da Huang W Sherman BT Lempicki RA Bioinformatics enrichment tools: paths toward the comprehensive functional analysis of large gene lists Nucleic acids research 2009 37 1 1 13 19033363 10.1093/nar/gkn923 PMC2615629

[B11] Razick S Magklaras G Donaldson IM iRefIndex: a consolidated protein interaction database with provenance BMC bioinformatics 2008 9 405 18823568 10.1186/1471-2105-9-405 PMC2573892

[B12] Turinsky AL Razick S Turner B Donaldson IM Wodak SJ Literature curation of protein interactions: measuring agreement across major public databases Database: the journal of biological databases and curation 2010 2010 baq026 21183497 10.1093/database/baq026 PMC3011985

[B13] Turner B Razick S Turinsky AL Vlasblom J Crowdy EK Cho E Morrison K Donaldson IM Wodak SJ iRefWeb: interactive analysis of consolidated protein interaction data and their supporting evidence Database: the journal of biological databases and curation 2010 2010 baq023 20940177 10.1093/database/baq023 PMC2963317

[B14] Mora A Donaldson IM iRefR: an R package to manipulate the iRefIndex consolidated protein interaction database BMC bioinformatics 2011 12 1 455 22115179 10.1186/1471-2105-12-455 PMC3282787

[B15] Kann MG Protein interactions and disease: computational approaches to uncover the etiology of diseases Briefings in bioinformatics 2007 8 5 333 346 17638813 10.1093/bib/bbm031

[B16] Oti M Brunner HG The modular nature of genetic diseases Clinical genetics 2007 71 1 1 11 17204041 10.1111/j.1399-0004.2006.00708.x

[B17] Badano JL Katsanis N Beyond Mendel: an evolving view of human genetic disease transmission Nature reviews Genetics 2002 3 10 779 789 10.1038/nrg910 12360236

[B18] Deakyne JS Mazin AV Fanconi anemia: at the crossroads of DNA repair Biochemistry Biokhimiia 2011 76 1 36 48 21568838 10.1134/s0006297911010068

[B19] Razick S Mora A Michalickova K Boddie P Donaldson IM iRefScape. A Cytoscape plug-in for visualization and data mining of protein interaction data from iRefIndex BMC bioinformatics 2011 12 388 21975162 10.1186/1471-2105-12-388 PMC3228863

[B20] The OMIM Morbid Map ftp://ftp.ncbi.nih.gov/repository/OMIM

[B21] Entrez Gene FTP Site ftp://ftp.ncbi.nih.gov/gene/README

[B22] Aranda B Achuthan P Alam-Faruque Y Armean I Bridge A Derow C Feuermann M Ghanbarian AT Kerrien S Khadake J The IntAct molecular interaction database in 2010 Nucleic acids research 2010 38 Database issue D525 531 19850723 10.1093/nar/gkp878 PMC2808934

[B23] Keshava Prasad TS Goel R Kandasamy K Keerthikumar S Kumar S Mathivanan S Telikicherla D Raju R Shafreen B Venugopal A Human Protein Reference Database--2009 update Nucleic acids research 2009 37 Database issue D767 772 18988627 10.1093/nar/gkn892 PMC2686490

[B24] Ruepp A Brauner B Dunger-Kaltenbach I Frishman G Montrone C Stransky M Waegele B Schmidt T Doudieu ON Stumpflen V CORUM: the comprehensive resource of mammalian protein complexes Nucleic acids research 2008 36 Database issue D646 650 17965090 10.1093/nar/gkm936 PMC2238909

[B25] Xenarios I Salwinski L Duan XJ Higney P Kim SM Eisenberg D DIP, the Database of Interacting Proteins: a research tool for studying cellular networks of protein interactions Nucleic acids research 2002 30 1 303 305 11752321 10.1093/nar/30.1.303 PMC99070

[B26] Bader GD Donaldson I Wolting C Ouellette BF Pawson T Hogue CW BIND–The Biomolecular Interaction Network Database Nucleic acids research 2001 29 1 242 245 11125103 10.1093/nar/29.1.242 PMC29820

[B27] Ceol A Chatr Aryamontri A Licata L Peluso D Briganti L Perfetto L Castagnoli L Cesareni G MINT, the molecular interaction database: 2009 update Nucleic acids research 2010 38 Database issue D532 539 19897547 10.1093/nar/gkp983 PMC2808973

[B28] Lynn DJ Winsor GL Chan C Richard N Laird MR Barsky A Gardy JL Roche FM Chan TH Shah N InnateDB: facilitating systems-level analyses of the mammalian innate immune response Molecular systems biology 2008 4 218 18766178 10.1038/msb.2008.55 PMC2564732

[B29] Hudson BG The molecular basis of Goodpasture and Alport syndromes: beacons for the discovery of the collagen IV family Journal of the American Society of Nephrology: JASN 2004 15 10 2514 2527 15466256 10.1097/01.ASN.0000141462.00630.76

[B30] Orchard S Kerrien S Abbani S Aranda B Bhate J Bidwell S Bridge A Briganti L Brinkman F Cesareni G Protein interaction data curation: the International Molecular Exchange (IMEx) consortium Nature methods 2012 9 4 345 350 22453911 10.1038/nmeth.1931 PMC3703241

[B31] Goldberg DS Roth FP Assessing experimentally derived interactions in a small world Proceedings of the National Academy of Sciences of the United States of America 2003 100 8 4372 4376 12676999 10.1073/pnas.0735871100 PMC404686

[B32] Berdiev BK Jovov B Tucker WC Naren AP Fuller CM Chapman ER Benos DJ ENaC subunit-subunit interactions and inhibition by syntaxin 1A American journal of physiology Renal physiology 2004 286 6 F1100 1106 14996668 10.1152/ajprenal.00344.2003

[B33] Stark C Breitkreutz BJ Chatr-Aryamontri A Boucher L Oughtred R Livstone MS Nixon J Van Auken K Wang X Shi X The BioGRID Interaction Database: 2011 update Nucleic acids research 2011 39 Database issue D698 704 21071413 10.1093/nar/gkq1116 PMC3013707

[B34] Brown KR Jurisica I Online predicted human interaction database Bioinformatics 2005 21 9 2076 2082 15657099 10.1093/bioinformatics/bti273

[B35] Pagel P Kovac S Oesterheld M Brauner B Dunger-Kaltenbach I Frishman G Montrone C Mark P Stumpflen V Mewes HW The MIPS mammalian protein-protein interaction database Bioinformatics 2005 21 6 832 834 15531608 10.1093/bioinformatics/bti115

[B36] Chautard E Fatoux-Ardore M Ballut L Thierry-Mieg N Ricard-Blum S MatrixDB, the extracellular matrix interaction database Nucleic acids research 2011 39 Database issue D235 240 20852260 10.1093/nar/gkq830 PMC3013758

[B37] Kanehisa M Goto S KEGG: kyoto encyclopedia of genes and genomes Nucleic acids research 2000 28 1 27 30 10592173 10.1093/nar/28.1.27 PMC102409

[B38] CRAN -The Comprehensive R Archive Networkhttp://cran.r-project.org/

[B39] Bioconductor -Open Source Software for Bioinformaticshttp://www.bioconductor.org/

[B40] OMIM Frequently Asked Questions (FAQs) http://omim.org/help/faq

[B41] MI Ontology Browser http://www.ebi.ac.uk/ontology-lookup/browse.do?ontName=MI

