# Supplementary figures and images for: A survey of protein interaction data and multigenic inherited disorders
Source: BMC Bioinformatics. 2013 Feb 11;14:47. doi: 10.1186/1471-2105-14-47 (PMC3598893; doi:10.1186/1471-2105-14-47)

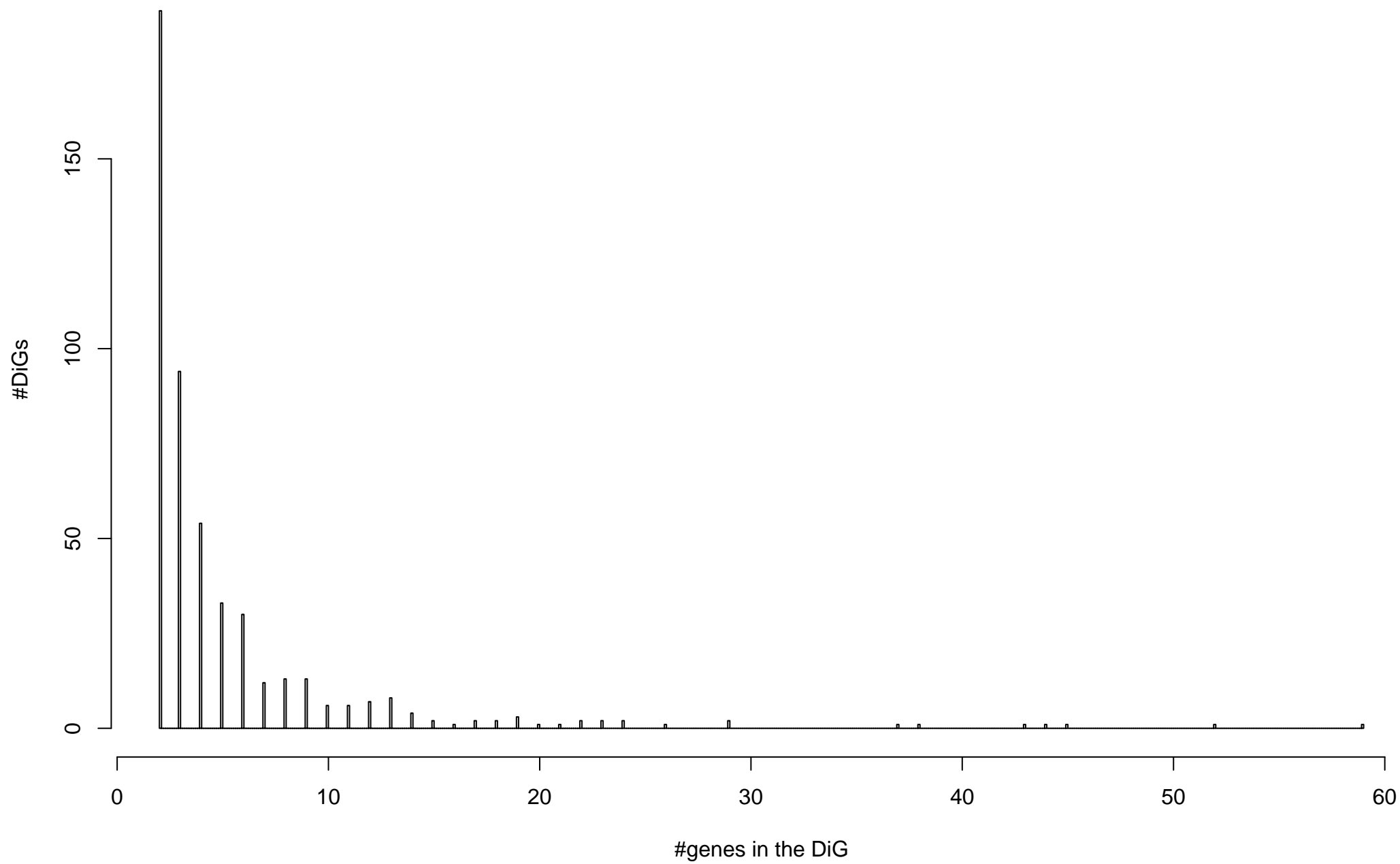

Supplement: Additional file 2 — Distribution of number of genes per disease group (DiG). [file 1471-2105-14-47-S2.pdf]
